# Vitamin D Status and Predictors of Hypovitaminosis D in Internationally Adopted Children

**DOI:** 10.1371/journal.pone.0158469

**Published:** 2016-09-29

**Authors:** Elena Chiappini, Francesco Vierucci, Francesca Ghetti, Maurizio de Martino, Luisa Galli

**Affiliations:** 1 Department of Health Sciences, Meyer Children University Hospital, University of Florence, Florence, Italy; 2 Pediatric Unit, S. Luca Hospital, Lucca, Italy; University of Padova, ITALY

## Abstract

**Objectives:**

To evaluate vitamin D status in internationally adopted children at first medical evaluation in Italy and to identify possible risk factors for hypovitaminosis D in this population.

**Methods:**

25-hydroxyvitamin D [25(OH)D] levels were analyzed in internationally adopted children consecutively recruited at one Italian Center between 2010 and 2014 as part of the first screening protocol. Demographic, clinical and laboratory data were prospectively collected. Serum 25(OH)D levels <10 ng/mL, <20 ng/mL, and <30 ng/mL were used to define severe vitamin D deficiency, vitamin D deficiency and hypovitaminosis D, respectively.

**Results:**

962 internationally adopted children (median age: 5.47 years; IQR:3.14–7.93) were included in the study. Median 25(OH)D level was 22.0 ng/mL (IQR:15.0–30.0 ng/mL); 710/962 (73.8%) children showed hypovitaminosis D (<30 ng/mL), 388/962 (40.3%) had vitamin D deficiency (<20 ng/dL), and 92/962 (9.6%) had severe vitamin D deficiency (<10ng/mL). No case of clinical rickets was observed. Hypovitaminosis D was particularly frequent (>90%) in children adopted from Ethiopia, Peru, India, Bulgaria and Lithuania. At multivariate analysis an increased risk of hypovitaminosis D was found to be associated with: age ≥ 6 years, time spent in Italy ≥ 3 months, blood sample taken in winter, spring or fall, compared to summer. Gender, ethnicity/continent of origin, tubercular infection, intestinal parassitosis and BMI-z-score < -2 were not associated with vitamin D status.

**Conclusion:**

Hypovitaminosis D is common in internationally adopted children, from all ethnic group. The evaluation of serum 25(OH)D level could be useful early after the adoption to promptly start vitamin D supplementation/treatment if needed.

## Introduction

Recent epidemiological studies have shown that hypovitaminosis D affects infants, children and adolescents worldwide [1–3]. A sufficient vitamin D status should be promoted during paediatric age to prevent the develop of nutritional rickets and to optimize bone health. Indeed, vitamin D not only plays a fundamental role in bone mass accrual, up to the acquisition of peak bone mass, but displays also several important extra-skeletal functions [4–6]. Sunlight exposure is the major source of vitamin D and contributes up to 90–95% of vitamin supply, while the number of foods naturally containing a substantial amount of vitamin D is very limited [7].

High rate of hypovitaminosis D in Italian children and adolescents living in Italy have been previously [8,9,10], suggesting that Italian children are at risk for this condition, as well as other European populations.

According to official data provided by the Italian Health Ministry, 42,048 children have been internationally adopted in Italy from November 16, 2000 to December 31, 2013 [11]. Adopted children should undergo an accurate medical screening at arrival since a high proportion may have experienced malnutrition, infectious diseases or other health problems in their original countries [12–13]. However, to date, really few studies have evaluated vitamin D status in internationally adopted children, reporting a prevalence of hypovitaminosis D ranging from 21% to 33% [14–15]. Moreover, some cases of nutritional rickets have been described in adopted children [16, 17]. The aims of this study were: 1) to evaluate the prevalence of hypovitaminosis D in a large cohort of internationally adopted infants, children and adolescents at their first medical evaluation in Italy; 2) to identify possible risk factors for hypovitaminosis D including age, gender, time spent in Italy, season of blood sample, continent of origin, body mass index (BMI), presence of other infectious disease such as parasitic or tuberculosis infection (TB).

## Methods

### Study population

All internationally adopted children (0–18 years) consecutively recruited at the Center for the internationally adopted children, Anna Meyer Children’s University Hospital, Florence, Italy in a 5 year period (January 2010-December 2014) underwent the internal operative protocol for the first screening and were enrolled in the study. The only exclusion criterium was the absence of 25(OH)D serum level results. Medical records were prospectively collected and entered into an electronic database (S1 Dataset). The demographic data collected included country of origin, age at adoption, age at first medical evaluation. Written informed consent to the study was obtained from all the parents of the enrolled children. The study was approved by the Ethics Committee for Human Investigation at Anna Meyer Children’s University Hospital.

### Screening protocol

According to and internal standard operative protocol used in our Hospital, and developed following the international recommendations [12], children underwent a clinical evaluation including an auxological evaluation (measuring length or height depending on age and weight were recorded are the mean of three measurements). Preadoption immunization records, including receipt of Bacille Calmete Guerin (BCG) vaccine and other vaccines were reviewed, and the presence of a BCG scar was noted. Eventual signs of rickets were recorded. At the first evaluation all the children underwent a venuputure and blood tests were performed including a dosage of serum 25-hydroxyvitamin D [25(OH)D] levels. All samples were measured in one laboratory that takes part in and meets the performance targets for the vitamin D external quality assessment scheme (DEQAS). The protocol did not include the evaluation of serum calcium, phosphate, parathormone or alkaline fosfatase.

A tuberculin skin test (TST) and Quantiferon Gold In tube assay (QFT-G-IT) were performed and a chest radiograph was executed if TST or QFT-G-IT were positive, according to international guidelines definitions [18]. Other blood tests executed included serologic tests for sevral infectious diseases (hepatitis B surface antigen and antibody, core antibody, hepatitis C, syphilis, *Toxocara canis* and human immunodeficiency virus). Finally, three stool samples were collected for the search for ova and parasites and for the antigen test for *Giardia lamblia*.

### Laboratory tests and Tuberculin skin test

All the other laboratory examinations were performed in the same laboratory at the Meyer Children Hospital, using standardized techniques and according to manufacturers’ instructions.

Serum 25(OH)D levels were determined by chemiluminescence enzyme-labeled immunometric assays using an IMMULITE 2000 Systems analyzer (Siemens, Gwynedd, UK). The intra- and inter-assays CV were <5% and <8%.

QuantiFERON-TB Gold In-TubeThe QFT-G-IT test was performed according to the manufacturer’s specifications. After subtracting the value from the negative control, the result was positive if, the antigen-dependent response was > = 0.35 IU, negative if the mitogen-induced response was > = 0.5 IU/mL and the antigen-dependent response was <0.35 IU/mL, and indeterminate if both mitogen-induced and antigen-dependent responses were below cutoff or mitogen-induced response was > 8 IU/mL.

Tuberculin Skin Test (TST) was administered by trained nurses dedicated to our Infectious Diseases Unit, and was performed according to the Mantoux method by injecting intradermally 5 tuberculin units (in 0.1 mL) of purified protein derivative into the volar surface of the forearm. The transversed skin induration was recorded (in millimeters) after 48–72 hours directly by a pediatrician of the Infectious Disease Unit. Following the American Academy of Pediatrics guidelines [18] a positive TST was defined as an induration size > = 10 mm as for children born in countries with a high prevalence of TB and who recently immigrated.

### Definitions

Length/height and BMI were expressed as z-score for age using the WHO Anthro software (World Health Organization, Geneva). Reduced length/height and BMI were defined as z-score < -2.

Hypovitaminosis D was defined as a 25(OH)D serum level < 30 ng/dL; Vitamin D deficiency was defined as a 25(OH)D serum level < 20.0 ng/mL; severe deficiency was defined as a level < 10 ng/mL; whereas levels ≥ of 30.0 ng/mL were considered sufficient [1].

Active TB diagnosis was assigned to any child with *Mycobacterium tuberculosis* cultured or detected by microscopy or molecular methods from sputum, gastric aspirate or other biologic samples [18]. Active TB diagnosis was also assigned to any child with clinical and radiological evidence of TB disease, and with either a history of exposure to an infectious case or a positive TST. In the absence of a recognized gold standard, latent tuberculosis diagnosis was assigned to any child with a positive TST and/or IGRA and no clinical, bacteriological or radiographic evidence of active TB [18].

## Statistical Analyses

Data were reported as median and interquartile range or absolute numbers and percentages. All continuous variables were not normally distributed thus the non parametric Mann-Whitney test was used to compare groups. Fisher’s exact test or Chi Square test were used to compare categorical variables, as appropriate. A multivariate logistic regression analysis was performed to investigate the association between presumed risk factor and severe vitamin D deficiency, vitamin D deficiency and hypovitaminosis D. All statistical analyses were carried out using the SPSS (Statistical Package of Social Sciences, Chicago, IL, USA) for Windows software program version 19.0. A p value < 0.05 was considered significant.

## Results

### Characteristics of the study population

Data from 962 internationally adopted children were collected and included in the study. Characteristics of the 962 study children (age range 0.29–16.18 years) are summarized in Table 1. At the time of medical evaluation none was receiving vitamin D supplementation and none had clinical sign of rickets. Most of the children originated from Europe (425/962;44.2%), followed from South America (206/962; 21.4%); Africa (174/962; 18.1%) and Asia (157/962;16.32%). We observed a 100% agreement between country of origin and ethnicity (i.e. all the children adopted from Asia displayed Asiatic ethnic traits, all the children from Europe were Caucasics, all the African children were Blacks and all the South American children were Hispanics).

**Table 1:** Characteristics of the 962 internationally adopted children included in the study.

	**Median (IQR)**
Age at blood evaluation, years	5.47 (3.14–7.93)
Age at adoption, years	5.23 (2.93–7.60)
Time spent in Italy before evaluation, days	72 (47–110)
Height/length, Z-score	-0.50 (-1.27; 0.39)
BMI, Z-score	-0.05 (-0.88; 0.72)
	**N (%)**
***Age***	
< 6 years	530/962 (55.09)
≥ 6 years	432/962 (44.91)
***Gender***	
Male	581/962 (60.40)
Female	381/962 (39.60)
***Time spent in Italy before evaluation***	
< 3 months	639/962 (66.42)
≥ 3 months	323/962 (33.58)
***Season of blood sample***	
Winter (Jan–Mar)	237/962 (24.64)
Spring (Apr–Jun)	241/962 (25.05)
Summer (Jul–Sep)	245/962 (25.47)
Fall (Oct–Dec)	239/962 (24.84)
***Continent of origin***	
Africa	174/962 (18.09)
South America	206/962 (21.41)
Asia	157/962 (16.32)
Europe	425/962 (44.18)

At auxological evaluation 7.28% (70/962) children had a BMI z score < -2. Screening for intestinal parassitosis or tuberculosis infection was positive in 291/962 (30.25%) and 46/962 (4.78%) children, respectively (2.08% were positive for both). Particularly, *Giardia lamblia* was documented in 168 children, *Toxocara canis* in 159 children, *Entamoeba* spp. in 82 children, *Strongyloides stercoralis* in 5 children, *Taenia solum* in 1 child. Twenty-nine children (3.01%) had more than one parasitic infection. Two children had active pulmonary tuberculosis and 44 had a latent tuberculosis infection. Considering other infections: 8 children were hepatitis B infected; 4 hepatitis A infected, 1 hepatitis C infected and none had HIV infection nor syphilis.

### Vitamin D status

The median 25(OH)D level was 22.0 ng/mL (IQR 15.0–30.0 ng/mL); 710/962 (73.8%) children showed hypovitaminosis D (<30 ng/mL), 388/962 (40.3%) had vitamin D deficiency (<20 ng/dL), and 92/962 (9.6%) had severe vitamin D deficiency (<10 ng/mL).

The prevalence of severe vitamin D deficiency, vitamin D deficiency and hypovitaminosis D in internationally adopted children according to the country of origin/ethnic group is summarized in Table 2. Hypovitaminosis D deficiency was particularly frequent (>90%) in children adopted from Ethiopia, Peru, India, Bulgaria and Lithuania.

**Table 2:** Vitamin D status in internationally adopted children divided for country of origin.

	Severe vitamin D deficiency, n (%)	Vitamin D deficiency, n (%)	Hypovitaminosis D, n (%)	Vitamin D sufficiency, n (%)
**Africa (n = 174)**				
Burkina Faso (n = 20)	1 (5.0)	12 (60.0)	17 (85.0)	3 (15.0)
Congo (n = 42)	2 (4.8)	13 (30.9)	28 (66.7)	14 (33.3)
Ethiopia (n = 90)	13 (14.4)	49 (54.4)	78 (86.7)	12 (13.3)
Other countries (n = 22)	0 (0.0)	3 (13.6)	14 (63.6)	8 (36.4)
**South America (n = 206)**				
Brazil (n = 43)	2 (4.6)	16 (37.2)	27 (57.1)	16 (37.2)
Chile (n = 43)	5 (11.6)	18 (41.9)	38 (88.4)	5 (11.6)
Colombia (n = 78)	2 (2.6)	25 (32.0)	58 (74.4)	20 (25.6)
Peru (n = 28)	5 (17.9)	19 (67.9)	25 (89.3)	3 (10.7)
Other countries (n = 14)	0 (0.0)	1 (7.1)	9 (64.3)	5 (35.7)
**Asia (n = 157)**				
China (n = 27)	0 (0.0)	5 (18.5)	20 (74.1)	7 (25.9)
Philippines (n = 14)	0 (0.0)	4 (28.6)	11 (78.6)	3 (21.4)
India (n = 55)	15 (27.3)	41 (74.5)	50 (90.9)	5 (9.1)
Vietnam (n = 37)	0 (0.0)	4 (10.8)	12 (32.4)	25 (67.6)
Other countries (n = 24)	4 (16.7)	9 (37.5)	13 (54.2)	11 (45.8)
**Europe (n = 425)**				
Bulgaria (n = 29)	9 (31.0)	18 (62.1)	26 (89.7)	3 (10.3)
Lithuania (n = 10)	2 (20.0)	4 (40.0)	9 (90.0)	1 (10.0)
Poland (n = 21)	0 (0.0)	6 (28.6)	14 (66.7)	7 (33.3)
Russia (n = 281)	29 (10.3)	113 (40.2)	198 (70.5)	83 (29.5)
Ukraine (n = 43)	2 (4.6)	12 (27.9)	29 (67.4)	14 (32.6)
Hungary (n = 26)	0 (0.0)	8 (30.8)	20 (76.9)	6 (23.1)
Other countries (n = 15)	1 (6.7)	8 (53.3)	13 (86.7)	2 (13.3)

Serum 25(OH)D levels and vitamin D status in relation to the presumed risk factors for hypovitaminosis D have been reported in Table 3. Serum 25(OH)D levels significantly differed by age. Children older than 6 years showed lower serum 25(OH)D levels and a higher prevalence of hypovitaminosis D compared with children younger than 6 years (Table 3).

**Table 3:** Serum 25(OH)D levels [median (IQR)] and vitamin D status related to presumed risk factors for hypovitaminosis D.

	n/tot (%)	25(OH)D, ng/mL	p	n/tot (%) with severe deficiency (<10 ng/dL)	p	n/tot (%) with deficiency (<20 ng/dL)	p	n/tot (%) with hypovitaminosis D (<30 ng/dL)	p
Entire sample	962/962 (100%)	22.0 (15.0–30.0)		92/962 (9.56)		388/962 (40.33)		710/962 (73.80)	
***Age***			**<0.0001**		0.226		**<0.0001**		**<0.0001**
< 6 years	530/962 (55.09)	24.0 (16.0–32.2)		45/530 (8.49)		183/530 (34.53)		354/530 (66.79)	
≥ 6 years	432/962 (44.91)	20.0 (13.0–26.7)		47/432 (10.88)		205/432 (47.45)		356/432 (82.41)	
***Gender***			0.819		0.823		0.271		0.231
Male	581/962 (60.40)	22.0 (15.0–29.0)		57/581 (9.81)		232/581 (39.93)		437/581 (75.22)	
Female	381/962 (39.60)	22.0 (14.0–31.0)		35/381 (9.19)		156/381 (40.94)		273/381 (71.65)	
***Time spent in Italy***			**<0.0001**		**0.009**		**0.007**		**<0.0001**
< 3 months	639/962 (66.42)	23.0 (16.0–32.0)		50/639 (7.82)		238/639 (37.24)		439/639 (68.70)	
≥ 3 months	323/962 (33.58)	20.0 (12.7–26.0)		42/323 (13.00)		150/323 (46.43)		271/323 (83.90)	
***Season of blood sample***			**<0.0001**		**<0.0001**		**<0.0001**		**<0.0001**
Winter (Jan–Mar)	237/962 (24.64)	15.0 (10.0–23.0)		52/237 (21.94)		158/237 (66.67)		202/237 (85.23)	
Spring (Apr–Jun)	241/962 (25.05)	20.0 (14.0–26.0)		21/241 (8.71)		108/241 (44.81)		205/241 (85.06)	
Summer (Jul–Sep)	245/962 (25.47)	31.0 (24.0–39.0)		2/245 (0.82)		29/245 (11.84)		113/245 (46.12)	
Fall (Oct–Dec)	239/962 (24.84)	22.0 (16.0–28.0)		17/239 (7.11)		93/239 (38.91)		190/239 (79.5)	
***Continent of origin***			0.595		0.366		0.207		0.103
Africa	174/962 (18.09)	21.0 (14.0–28.2)		16/174 (9.20)		77/174 (44.25)		137/174 (78.74)	
South America	206/962 (21.41)	22.0 (16.0–29.0)		14/206 (6.80)		79/206 (38.35)		157/206 (76.21)	
Asia	157/962 (16.32)	23.0 (13.5–33.0)		19/157 (12.1)		63/157 (40.13)		106/157 (67.52)	
Europe	425/962 (44.18)	23.0 (14.0–30.0)		43/425 (10.12)		169/425 (39.76)		310/425 (72.94)	
***BMI Z-score***			0.400		0.897		0.483		0.638
< -2	70/962 (7.28)	22.0 (14.0–31.0)		7/70 (10.07)		31/70 (44.29)		50/70 (71.42)	
≥ -2	892/962 (92.72)	21.0 (14.0–28.0)		85/892 (9.52)		357/892 (40.02)		660/892 (73.99)	
***Parassitosis***			0.544		0.812		0.312		0.151
Positive	291/962 (30.25)	22.0 (14.0–29.0)		29/291 (9.97)		120/291 (41.24)		224/291 (76.98)	
Negative	671/962 (69.75)	23.0 (15.0–30.0)		63/671 (9.39)		268/671 (39.94)		486/671 (72.43)	
***TB infection***			0.189		0.436		0.378		0.228
Positive	46/962 (4.78)	20.5 (11.7–26.0)		6/46 (13.04)		21/46 (45.65)		38/46 (82.61)	
Negative	916/962 (95.22)	22.0 (15.0–30.0)		86/916 (9.39)		367/916 (40.07)		672/916 (73.36)	

Children that had spent at least 3 months in Italy before performing blood examination showed lower serum 25(OH)D levels and higher prevalence of severe vitamin D deficiency and hypovitaminosis D compared to children evaluated within 3 months from their arrival in Italy (Table 3).

In a subanalysis considering the continent of origin, lower 25(OH)D levels were confirmed in subjects that had spent at least 3 months in Italy adopted from Africa [19.0 (12.0–25.0) ng/mL ≥ 3 months vs 24.0 (16.0–31.0) ng/mL < 3 months, p = 0.004], South America [19.5 (13.0–26.0) ng/mL ≥ 3 months vs 23.0 (18.0–32.0) ng/mL < 3 months, p = 0.002], and Asia [17 (10.25–24.0) ng/mL ≥ 3 months vs 26.0 (16.25–34.75) ng/mL < 3 months, p = 0.001] but not in those adopted from European countries [22 (14–27) ng/mL ≥ 3 months vs 23.0 (14–31.75) ng/mL < 3 months, p = 0.220].

Seasonal differences in vitamin D status were evident. Particularly, serum 25(OH)D levels were in the range of sufficiency only in summer while subjects evaluated during winter had median serum 25(OH)D levels in the range of vitamin D deficiency (Table 3). Vitamin D levels were not significant different considering gender, continent of origin, BMI Z-score and parassitosis or TB infection (Table 3).

Multiple logistic regression analyses for the presumed predictors of vitamin D status is showed in Table 4. The season of blood sample was the only factor significantly associated with an increased adjusted odds ratio (aOR) of severe vitamin D deficiency. Indeed, subjects who were evaluated in winter, spring or fall had an aOR of severe vitamin D deficiency 38.38, 12.11, or 9.66 times higher than those who were evaluated in summer, confirming marked seasonal variation in vitamin D status. Seasonality was associated also with increased OR of vitamin D deficiency and hypovitaminosis D. Individuals older than 6 year had an aOR of vitamin D deficiency and hypovitaminosis D 1.87 and 2.50 times higher than subjects younger than 6 years, respectively. Finally, children who had spent at least 3 months in Italy before performing blood evaluation showed an OR of 1.77 of having hypovitaminosis D. Gender, continent of origin and BMI Z-score were not associated with an increased aOR for hypovitaminosis D.

**Table 4:** Multiple logistic regression for presumed risk factors for severe vitamin D deficiency, vitamin D deficiency and hypovitaminosis D.

	Severe vitamin D deficiency				Vitamin D deficiency				Hypovitaminosis D			
	25(OH)D < 10 ng/mL				25(OH)D < 20 ng/mL				25(OH)D < 30 ng/mL			
	B (SE)	aOR	95%CI	p	B (SE)	aOR	95%CI	p	B (SE)	aOR	95%CI	p
*Gender*												
(female vs male	0.07 (0.24)	1.07	0.67–1.73	0.771	0.11 (0.15)	1.11	0.83–1.50	0.474	-0.22 (0.17)	0.81	0.58–1.12	0.203
*Age*												
≥ 6 years vs < 6	0.23 (0.24)	1.26	0.79–2.01	0.340	0.63 (0.15)	1.87	1.39–2.52	**<0.0001**	0.92 (0.18)	2.50	1.77–3.53	**<0.0001**
*Time spent in Italy*												
≥ 3 months vs < 3	0.44 (0.24)	1.56	0.97–2.49	0.065	0.07 (0.16)	1.07	0.78–1.48	0.656	0.57 (0.20)	1.77	1.21–2.61	**0.004**
*Season blood sample*												
Summer	2.27 (0.75)	1 (reference)	2.20–42.38	**0.003**	1.60 (0.24)	1 (reference)	3.07–7.94	**<0.0001**	1.59 (0.22)	1 (reference)	3.20–7.47	**<0.0001**
Fall	3.65 (0.73)	9.66	9.11–161.67	**<0.0001**	2.79 (0.25)	4.94	9.94–26.64	**<0.0001**	1.88 (0.24)	4.89	4.10–10.48	**<0.0001**
Winter	2.49 (0.75)	38.38	2.79-52-45	**0.001**	1.78 (0.24)	16.27	3.69–9.48	**<0.0001**	1.91 (0.23)	6.56	4.31–10.71	**<0.0001**
Spring		12.11				5.92				6.79		
*Continent*												
not-Europe vs Europe	0.01 (0.24)	1.00	0.63–1.60	0.988	0.19 (0.15)	1.21	0.90–1.63	0.204	0.27 (0.17)	1.31	0.94–1.83	0.105
BMI Z-score												
(< -2 vs ≥ -2)	-0.20 (0.53)	0.81	0.29–2.28	0.698	-0.01 (0.35)	0.99	0.50–1.96	0.970	-0.22 (0.39)	0.80	0.38–1.72	0.573
	χ^2^ = 91.06, p<0.0001; Cox R^2^ = 0.090; Nagelkerke R^2^ = 0.193;Hosmer and Lemeshow test p = 0.663				χ^2^ = 197.66, p<0.0001; Cox R^2^ = 0.186; Nagelkerke R^2^ = 0.251;Hosmer and Lemeshow test p = 0.223				χ^2^ = 188.02, p<0.0001; Cox R^2^ = 0.178; Nagelkerke R^2^ = 0.260;Hosmer and Lemeshow test p = 0.099			

Note. Adjusted for year of blood sample. C.I.: Coefficient Interval

## Discussion

Recent epidemiological studies showed a high prevalence of vitamin D deficiency in infants, children, and adolescents in many countries around the world [2]. To our knowledge, this is the largest pediatric series assessing vitamin D status and the prevalence of hypovitaminosis D in internationally adopted children. Our results demonstrated a high prevalence (about 76%) of hypovitaminosis D in this population. In multivariate analyses increased risk of hypovitaminosis D was associated with: age ≥ 6, time spent in Italy ≥ 3 months before medical evaluation and blood samples taken in winter, spring or fall compared to summer. Conversely, gender, continent of origin, and BMI-z-score < -2 were not associated with increased vitamin D status.

Previous epidemiological studies reported a high and similar rate of hypovitaminosis D in Italian children and adolescents living in Tuscany (latitude 43–44°N) [19–21], suggesting that Italian children are also at risk of poor vitamin D status. Serum 25(OH)D levels of Italian children were influenced by sun exposure, seasonality, age, adiposity, and ethnicity. Particularly, Franchi *et al*. evaluated 1,374 children living in the north of Italy (latitude 45°N) confirming that ethnicity was a strong predictor of 25OH)D levels [22]. Results confirmed a high prevalence of hypovitaminosis D in Caucasian, African, North African, and Indian children living in Italy (74.8%, 81.2%, 89.7%, and 76.0%, respectively) [22]. Thus, we can speculate that a spontaneous recovery of vitamin D status is unlikely in an adopted child arrived in Italy with vitamin D deficiency or insufficiency, independently from their ethnicity.

We found that children evaluated 3 months or more after their arrival in Italy had lower serum 25(OH)D levels with an increased aOR of hypovitaminosis D of 1.77 times. There are several possible explanations for this result. After the adoption, children can modify their modalities of sun exposure in comparison to their previous habits, for example using sunscreens. The time spent in Italy was a predictor of vitamin D status in children adopted from Africa, South America and Asia but not from Europe, probably because children adopted from European countries lived at higher latitude in the north hemisphere, with consequent ineffective skin vitamin D synthesis most of the year. On the contrary many African, South American, and Asian countries are at lower latitude than Italy. Secondly, adopted children usually had a rapid recovery in weight gain [23] with a possible sequestration of vitamin D in adipose tissue [24]. Finally, vitamin D supplementation was not started until clinical and biochemical evaluation was performed. Our results reinforce the importance of an early evaluation of serum 25(OH)D levels in internationally adopted children to start the adequate strategy of vitamin D supplementation/treatment, avoiding further deterioration of vitamin D status.

Two recent studies evaluated vitamin D status in internationally adopted children at arrival in USA. Fuglestad et al. evaluated 42 infants (age 8–18 months) within one month of arrival, showing an overall prevalence of hypovitaminosis D of 21%, a rate lower than in our series. However, a large percentage of infants adopted from Post-Soviet States (mainly Russia) had hypovitaminosis D (64%), similarly to our findings [15]. Gustafson et al. evaluated vitamin D status in 160 children (age 4 months-17.8 years) within the first 6 months after adoption and showed a prevalence of hypovitaminosis D of 33% [14]. Children adopted from eastern Europe/Russia showed the highest prevalence of hypovitaminosis D (48%) also in this study. The different prevalence of hypovitaminosis D reported among studies may partly be due to differences in the enrolled population of adopted children such as age, sun exposure before adoption (length of institutionalization, latitude of the country of origin, skin phenotype), elapsed time from arrival to clinical evaluation, and possible vitamin D supplementation started before dosing 25(OH)D levels.

We found that age and season of blood sample significantly influenced vitamin D status of adopted children. Older children may have spent longer time in institution, with consequent reduced sun exposure, according to Gustafson et al. [14]. Unfortunately, information regarding the time spent in orphanages was not available in our study.

Season of blood sample was strongly related to vitamin D status. Particularly, winter or spring months were associated to a significant increased risk for severe vitamin D deficiency, a condition possibly associated with nutritional rickets. Most of our study children were resident in high-latitude countries in the north hemisphere, where sun-derived vitamin D synthesis is not effective most of the year. Moreover, we had previously demonstrated that no vitamin D is produced in Tuscany (latitude 43°N) during late fall, winter and the beginning of spring as a result of sun exposure [2]. Thus, serum 25(OH)D evaluation is particularly important in children arrived in Italy during winter and spring period.

In previous studies in Italian Children [20,21], an inverse correlation among BMI and 25(OH)D levels were observed. This findings may be due to the fact that vitamin D is a fat-soluble vitamin, possibly stored in the adipose tissue, leading to a reduced bioavailability in obese children [24]. On the other hand, in our study children with reduced BMI Z-score did not show lower 25(OH)D levels, probably because they have scarce adiposity with consequent increased bioavailability of vitamin D. Thus, reduced 25(OH)D should not be considered as a sign of malnutrition, also considering that foods are naturally poor in vitamin D [7].

Finally, in our study, gender, continent of origin, TB infection or parassitosis were not related to serum 25(OH)D levels. These results are consistent with those reported by Gustafson et al. that confirmed that birth country, TB infection and parasitic infections did not affect vitamin D status [14]. We previously demonstrated a relation between TB disease and vitamin D status [25–26]; however it should be noticed that in our study only two children has active TB disease, possibly explaining the discrepancy with respect to previous results.

A previous study evaluated health problems of 136 internationally adopted children arrived in Italy between April 2002 and May 2006 (age 12 months-15 years) [17]. Similarly to our results, auxological evaluation showed growth delay in a significant percentage of children (height and weight Z-score for age ≤ -2 in 19.1% and 18.4% of the children, respectively). Screening for infectious diseases showed latent TB in 9 (6.6%) children and intestinal parassitosis in 12 (8.8%) subjects. Clinical sign of rickets were present in 21 children (15.4%), but serum 25(OH)D levels were not evaluated. Of these, 3 individuals showed radiographic skeletal alterations compatible with florid rickets [17]. Other few cases of vitamin D deficiency rickets have been reported in adopted children: Hostetter et al. evaluated 293 internationally adopted children describing 1 case of rickets [27], Reeves et al. described nutritional rickets in 3 children adopted from the former Soviet Union [16], and Miller et al. reported 1 case of rickets among 452 children adopted from China [28] and another case among 103 children adopted from Guatemala [29]. The two previously cited studies that evaluated vitamin D status in adopted children did not report any case of rickets [14,15].

In our study none of the adopted children showed overt clinical signs of rickets. This finding may be partly explained considering that infants (< 1 year) enrolled in our study, the population at higher risk of rickets, showed adequate vitamin D status with median serum 25(OH)D levels in the range of sufficiency, probably because institutionalized infants are more prone to be fed with vitamin D-fortified formula rather than breastfed. Moreover, we chose not to perform wrist x-ray in the absence of overt clinical signs of rickets to avoid radiological exposure but possibly misleading initial cases of rickets.

Our cross sectional study had some limitations. Adopted children spent variable time in Italy before our evaluation, possibly influencing the results (more than 30% of the children spent more than 3 months in Italy without vitamin D supplementation). We did not have serial 25(OH)D determinations in these children or follow-up data. Finally, we could not evaluate other presumed risk factors of hypovitaminosis D, as time spent in institution or diet type and sun exposure before adoption.

## Conclusions

Hypovitaminosis D was common in internationally adopted children. However, despite a substantial prevalence of severe vitamin D deficiency, no case of nutritional rickets was observed. The evaluation of serum 25(OH)D levels is recommended early after the adoption, particularly in older children and in subjects arrived in Italy during winter and spring period, to promptly start vitamin D supplementation/treatment if needed.

## Supporting Information

S1 Dataset (XLS)
